# “WHEN WE ARRIVED IN THIS COUNTRY, WE WERE ALREADY VERY OLD”: HEALTH AND AGING IN WISCONSIN’S HMONG REFUGEE COMMUNITY

**DOI:** 10.1093/geroni/igad104.1231

**Published:** 2023-12-21

**Authors:** Michal Engelman, Maichou Lor, Mai Xiong, Tou Lee, Casper Vang

**Affiliations:** University of Wisconsin-Madison, Madison, Wisconsin, United States; University of Wisconsin-Madison, Madison, Wisconsin, United States; University of Wisconsin-Madison, Madison, Wisconsin, United States; University of Madison - Wisconsin, Madison, Wisconsin, United States; University of Wisconsin-Madison, Madison, Wisconsin, United States

## Abstract

The Hmong arrived in Wisconsin as refugees following wars in Vietnam and Laos, and are the state’s largest Asian American group. They have a disadvantaged socioeconomic profile and face high rates of trauma-linked mental illness. Due to limited English proficiency, low literacy, and a lack of familiarity with and trust in research, the Hmong are underrepresented in existing data sources, which fold them into a pan-ethnic Asian-American group considered exceptionally successful. To address this gap, the Diversity, Inclusion, and Aging in the Midwest: Opportunities for New Directions with Wisconsin’s Hmong Community (DIAMOND-Hmong) project has engaged Hmong older adults in a research study designed to document their unique life experiences and social determinants of health. Drawing on semi-structured life history interviews with 40 older Hmong men and women in Wisconsin, we describe participants’ experiences during wars, forced relocations, and refugee camp spells, as well as the challenges and rewards of migrating to and aging in the U.S. Using a constructionist grounded theory approach, we show that participants’ narratives link life-course hardships with physical and mental health challenges, generating a historically and culturally-specific delineation of trauma as both individual and collective experiences. Our analysis situates individual trauma within broader geopolitical and institutional circumstances and demonstrates that familial and communal ties – and their absence – are sources of both tension and resilience in this population. Our findings point to the importance of a trauma-informed approach to health assessment for the Hmong, and the role of memory and storytelling in their healing process.

